# Transcriptome view of a killer: African swine fever virus

**DOI:** 10.1042/BST20191108

**Published:** 2020-07-29

**Authors:** Gwenny Cackett, Michal Sýkora, Finn Werner

**Affiliations:** RNAP lab, Institute for Structural and Molecular Biology, Division of Biosciences, University College London, Gower Street, London WC1E 6BT, U.K.

**Keywords:** African swine fever virus, gene expression and regulation, RNA polymerase, RNA sequencing, transcription, virology

## Abstract

African swine fever virus (ASFV) represents a severe threat to global agriculture with the world's domestic pig population reduced by a quarter following recent outbreaks in Europe and Asia. Like other nucleocytoplasmic large DNA viruses, ASFV encodes a transcription apparatus including a eukaryote-like RNA polymerase along with a combination of virus-specific, and host-related transcription factors homologous to the TATA-binding protein (TBP) and TFIIB. Despite its high impact, the molecular basis and temporal regulation of ASFV transcription is not well understood. Our lab recently applied deep sequencing approaches to characterise the viral transcriptome and gene expression during early and late ASFV infection. We have characterised the viral promoter elements and termination signatures, by mapping the RNA-5′ and RNA-3′ termini at single nucleotide resolution. In this review, we discuss the emerging field of ASFV transcripts, transcription, and transcriptomics.

## Introduction to African swine fever

The emergence of African Swine Fever (ASF), caused by ASFV, was first documented almost 100 years ago in Kenya after the disease transferred from warthogs to domestic pigs, which developed a highly lethal haemorrhagic fever; unfortunately ASFV remains endemic in Sub-Saharan Africa [1]. The infected warthogs display minimal or no clinical symptoms. *Ornithodoros* (soft) ticks can act as a biological vector between warthogs and/or pigs, though direct pig-to-pig infection is the most frequent cause of disease propagation in an agricultural context [2]. There have been five documented incidents of cross-continental dispersal of ASFV, with subsequent spread among domestic pigs and wild boars. Most recently, ASFV spread to Georgia in 2007 [3], and further into Europe and China in 2018 [4], followed by additional outbreaks across South-East Asia [5]. In 2019 alone, ASF disease-related deaths and culling reduced the global farmed pig population by a quarter.

Despite extensive study of ASFV and its damaging impact, there are still no approved treatments or vaccines available. Research on attenuated virus-based vaccines, carrying deletions of virulence-associated genes, show promise, but have not yet been exploited commercially. Nor is it clear whether they can be developed and applied in time to circumvent further demise of domesticated pig populations [6].

The genomes of several ASFV strains have been sequenced and mapped; most molecular biological research has been carried out on the attenuated ASFV strain BA71V, derived from its highly virulent parental strain BA71, via adaptation to infection of *Vero* tissue culture cells [7,8]. Other noteworthy virulent ASFV strains include ASFV Georgia 2007 [3], Hungarian ASFV isolate 2018 [9], and Chinese ASFV Heilongjiang, 2018 (Pig/HLJ/18) [10] isolates. Most differences between the genomes of virulent and attenuated lab strains reside in pathogenicity islands, often encoding genes belonging to multigene families (MGF) whose function remain poorly understood [11].

## Introduction to the ASFV transcription system

A major limitation for developing effective antiviral treatments for ASFV is our limited knowledge of the molecular mechanisms of viral DNA replication and gene expression, i.e. transcription and translation. Much has been extrapolated from the paradigms of well-studied *Poxviridae* members including Vaccinia virus (VACV), or inferred from the eukaryotic Pol II transcription system [12,13]. Both VACV and ASFV have 170–200 kilobase double-stranded-DNA genomes, replicate in the host cytoplasm, and share similarities with other NCLDV families. As the name implies, Nucleocytoplasmic large DNA viruses (NCLDVs) replicate in the cytoplasm, and can include a nuclear replication stage [14–18]. Though, there is no persuasive evidence for a nuclear stage of ASFV, which appears more akin to *Poxviridae* that exclusively replicate in the cytoplasm [19]. The cytoplasmic localisation of ASFV prevents access to the host transcription machinery within the nucleus. This necessitates that ASFV encodes, and carries in the virions, all factors required to express and process transcripts during early infection: including the viral RNAP, transcription factors, viral capping enzyme and polyA polymerase [20–22]. In support of this notion, soluble extracts of ASFV particles are fully transcription competent *in vitro* [23]. This likely reflects the evolutionary selection pressures at work in the virus-host arms race. In essence, the nucleus of the host cell provides a protective environment for its genome and keeps the RNA polymerase (RNAP) and transcription factors in an isolated subcellular environment not readily available for the sequestration and subjugation by viruses. Transcription machineries encoded by the viral genome in turn, provide NCLDVs like ASFV with a strong selective advantage because they have become transcriptionally self-sufficient. This host-factor independence may also enable ASFV to propagate across pig species, and its evolutionary-distant tick vectors.

## ASFV RNA polymerase and transcription factors

**Figure 1.**
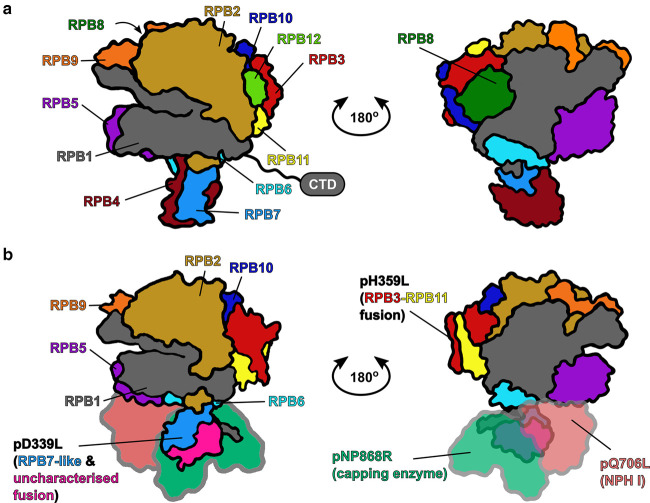
RNA polymerases from ASFV is closely related to eukaryotic Pol II. (**a**) Diagrammatic representation of eukaryotic Pol II from *S. cerevisiae* drawn from PDB: 6GYK. CTD refers to the ‘carboxy terminal domain’. (**b**) The NCLDV-RNAP represented by ASFV-RNAP*.* This diagrammatic model was drawn from PHYRE2 homology models [114] for ASFV homologs of Pol II subunits, mapped onto the Pol II structure using UCSF Chimera [115]. Homologous RNAP subunits are colour-coded and annotated, please note that the RPB3 and 11 subunits are fused in both ASFV and VACV, and ASFV-RPB7 contains an extended C-terminus, with no homology to characterised proteins [24,28]. Additionally, beyond canonical Pol II subunits, NCLDV-RNAPs also include, based on structurally characterised VACV-RNAP, a stably integrated mRNA capping enzyme (NP868R, highlighted in pale green) and termination factor (Q706L, in pale red) [28,29].

ASFV-RNAP shares many commonalities with eukaryotic Pol II transcription systems [12,13,15,24]: encoding homologs of 9 (of 12) Pol II subunits, though lacking the RPB1 carboxy terminal domain, and encodes homologs of general transcription initiation factors TBP and TFIIB [12,20,23,25], as well as transcript cleavage/elongation factor TFIIS (Figure 1 and Table 1). NCLDVs such as ASFV additionally encode virus-specific transcription initiation factors without any eukaryotic homologs that direct transcription from distinct viral promoters [13,26]. The exact origin of the NCLDV-RNAPs is still a subject of debate: it remains unclear whether they predate eukaryotic RNAPs, are derived from one of the Pols I, II, and III, or perhaps precede their divergence [21,24,27]. The transcription system of VACV is more extensively studied than ASFV [13]: the structure of VACV-RNAP has been solved by cryo-EM [28,29] and the mechanism and regulation of VACV transcription has been characterised using biochemical and NGS-based approaches [30–34]. ASFV utilises a more Pol II-like system compared with VACV, which lacks RNAP subunit RPB9, and TFIIB.

**Table 1:** Evolutionary conservation of RNA polymerase subunits and general transcription factors

Pol II	ASFV Gene	VACV-RNAP	VACV Gene
RPB1	NP1450L [25]	Rpo147 [96]	J6R
RPB2	EP1242L [25]	Rpo132 [97]	A24R
RPB3-11 fusion	H359L [8]	Rpo35 [28,98]	A29R
RPB5	D205R [8,99]	Rpo22 [96]	J4R
RPB6	C147L [8,100]	Rpo19 [101]	A5R
RPB7	D339L [8]	Rpo18 [102]	D7R
RPB9	C105R [7]	-	-
RPB10	CP80R [8]	Rpo7 [103]	G5.5R
-	-	Rap94 [28,98]	H4L
Factor	ASFV gene	VACV gene	Function
VETF-L	G1340L [21]	A7L [28,104,105]	Initiation
VETF-S	D1133L [15,74,106,107]	D6R [28,104,105]	Initiation, ATPase activity
TBP	B263R [108]	A23L [109]	Initiation
TFIIB	C315R [21]	-	Initiation, RNAP-recruitment
TFIIS	I243L [35]	E4L (Rpo30 subunit) [110]	Elongation, release of arrested RNAP
NPH I	Q706L^1^	D11L [28,111]	Termination, chromatin
VTF/CE	NP868R [22]	D1R & D12L	Capping enzyme

The relationship between eukaryotic and NCDLV transcription systems from ASFV and VACV is apparent from conservation of Pol II subunit homologs, as well as core general transcription factors that guide Pol II function.

1 Q706L (Accession: NP_042814.1) was identified as a significant hit from PSI-BLAST [112,113] searching with VACV NPH I protein sequence (Query coverage: 84%, E-value: 4 × 10^−16^). The putative predicted functions of transcription factors are indicated. Note that the mRNA capping enzyme (VTF/CE) and termination factor (NPH I) is possibly stably integrated in the VACV-RNAP as shown in Figure 1.

## Discrete stages of viral gene expression during infection

Although ASFV and VACV share similarities, it is not clear whether ASFV transcription follows the VACV paradigm for transcription factor utilisation, molecular mechanisms and temporal regulation during infection. For example, VACV uses a cascade-like mechanism of viral gene expression in which the expression of promoter-specific transcription factors is required for expression of later genes [13]. It is thought that ASFV follows the four canonical stages of viral gene expression during infection: immediate early, early, intermediate, and late, though discriminating between these stages is nontrivial. The stages of infection in ASFV-BA71V have been deconvoluted by treating cells with combinations of replication- and/or translation inhibitors and the expression of a selection of genes studied using primer extension and S1 nuclease mapping [12,13,35–58]. While chemical intervention is convenient, it is not without caveats due to possible pleiotropic effects of inhibitors. These studies have provided evidence for distinct immediate early, early and late (post-replicative) gene expression stages, thought to correspond to times points prior to viral proteins synthesis, and before- and after replication, respectively. Two ASFV genes, I226R and I243L, have been shown to generate several mRNA species that are expressed during multiple stages of infection; these are under the control of independent promoters [47]. Two of the mRNA species follow the canonical intermediate pattern of expression i.e. transcription immediately following DNA replication. However, intermediate gene expression has not been analysed genome-wide and it is not clear whether genes other than I226R and I243L fall within this category. Whereas, intermediate gene expression has been persuasively demonstrated in VACV by utilising combinations of a DNA replication inhibitor and conditionally regulated intermediate/late transcription factors [59].

## The genome-wide, transcriptomic view of transcription

The first genome-wide view of ASFV transcription [58] revealed that almost all ASFV genes are actively transcribed. This is especially pronounced during late infection when the levels of all transcripts are higher, possibly due to increased genome copy number as consequences of ongoing virus replication. Highly expressed genes were also well correlated with highly abundant proteins identified in proteomic studies of ASFV-infected cells and in virus particles [20,60]. It is important to note, that early and late gene classification relies on achieving near 100% synchronicity of infection of all cells, and if it is not met, it could result in some contamination of late with early transcripts. Though it is more likely long half-lives of early mRNAs may create false positive signals among bona fide late genes, since RNA-seq analyses reflect RNA abundance — the sum of synthesis and decay. Classification difficulties aside, early and late stage expression are not necessarily mutually exclusive. Almost a quarter of ASFV mRNAs can be detected in significant amounts at both time-points, perhaps hinting at multi-stage expression of genes under the control of more than one promoter. Another interesting feature of ASFV transcripts is the presence and variation of the length of 5′ and 3′ untranslated regions (UTRs) [58], a feature that in cellular mRNAs often is associated with posttranscriptional regulation by e.g. miRNAs.

## NGS technology — mapping ASFV mRNAs

ASFV transcriptomics is a budding field with an array of techniques that can be utilised to investigate transcription from initiation to termination. A robust method to evaluate mRNA abundance is RNA-seq, which provides a good ‘first glance’ of a transcriptome, but which is often poor at discerning the precise 5′ and 3′ termini of mRNAs. ASFV mRNAs are modified with a 5′-Cap (m^7^GpppA^m^) and 3′-poly(A) tail [23], which allow the use of more specialised techniques including Cap Analysis Gene Expression sequencing (CAGE-seq) [61] and 3′ RNA-seq [62] to map the 5′ and 3′ nucleotides of transcripts, respectively [58]. Another cutting edge method recently applied to the ASFV transcriptome is Nanopore sequencing [63], which is able to generate long reads of individual mRNA transcripts and thus evaluate isoforms and variations (of e.g. mRNA length) in a population of transcripts. Viral genomes like that of ASFV are tightly packed with very short intergenic regions, and in cases where transcription termination is not 100% efficient, transcription elongation complexes ‘read through’ the 3′ end of a gene into the downstream gene. Terminator read-through creates problems with the assignment of early and late promoters in RNA-seq but not CAGE-seq, as the latter specifically maps the capped 5′ end of mRNAs [32,58].

## Transcripts expressed during early and late infection

Both CAGE-seq and RNA-seq have been applied to analyse and quantify transcript levels of ASFV genes during an infection time course [58]. Using RNA-seq, Jaing et al. [64] characterised ASFV transcripts in blood of pigs infected with either low-pathogenicity OUR T88/3 strain or the highly pathogenic Georgia 2007/1 (GRG) strain. One of the key findings of this analysis is that ASFV gene expression levels varied substantially between individual GRG-infected animals. While tissue culture systems cannot inform directly on effects of ASFV on the whole animal, they have distinct advantages over animal model systems, by providing a rigor of experimentation conditions in terms of reproducibility, homogeneity and synchronicity of infection. Our recent analysis applied both CAGE-seq and RNA-seq approaches to quantify gene expression levels during early (5 h) and late (16 h) infection of *Vero* cells with ASFV-BA71V, providing insight into temporal gene expression of known and novel viral genes [58].

**Figure 2.**
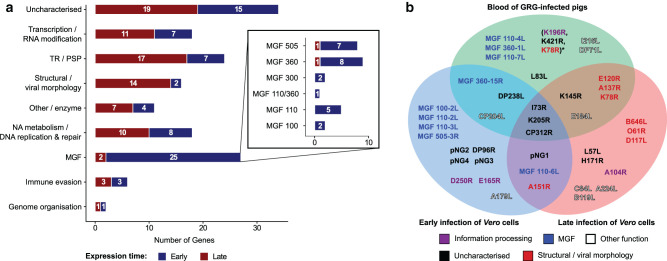
Temporal ASFV gene expression. (**a**) Differentially expressed genes during early and late infection of *Vero* cells with ASFV strain BA71V are categorised according to function: early (down-regulated) and late (up-regulated) genes are indicated in blue and red, respectively. (**b**) Venn diagram of highest 17 expressed ASFV genes detected in the blood of GRG-infected pigs using RNA-seq by Jaing et al. [64]. The two lower sectors correspond to 20 genes most expressed during early (5 h, left) and late (16 h, right) infection of *Vero* cells, regardless of differential expression, characterised by CAGE-seq [58]. Genes are colour-coded according to their predicted functional group (as in (**a**)): the group ‘information processing’ is a combination of ‘Transcription/RNA modification’, NA metabolism/DNA replication & repair’ and Genome organisation’.

CAGE-seq analysis of ASFV-BA71V-infected *Vero* cells identified 149 differentially expressed genes (DEGs). These were classified as either early or late based on statistically significant differential expression between 5 hours and 16 h post-infection, without use of chemical inhibitors: 65 genes were classified as early genes, 84 as late, and 7 genes were not differentially expressed. In comparison, RNA-seq analyses identified 47 early genes, 56 late, and 51 showed no significant differential expression. However, both independent techniques showed a significant correlation between the detected DEGs. Classification of DEGs into functional groups (Figure 2a) confirmed early expression of genes encoding proteins involved in genome replication and late transcription (including TBP homologue B263R), and multigene families (MGFs) associated with evasion of the host immune response [65]. Late expressed genes include structural proteins - required to form new viral particles, and early transcription factors packaged in virions [20,66,67].

The genome-wide ASFV expression profiles by Jaing et al. [64] and Cackett et al. [58] highlight a number of well conserved genes that are highly expressed throughout infection, thereby make interesting targets for vaccine development. Unfortunately, their molecular function remains unknown (Figure 2b), though some gene product immunogenicities have been investigated in infected pig models: CP312R and K205R, but not I73R, were effective antigens inducing an immune response [68,69]. These genes are promising for further characterisation and pharmaceutical targeting, given their clear importance during infection and lack of homology to host proteins.

As mentioned above, high levels of early transcripts were detected during late infection [58]. Without detailed knowledge of ASFV mRNA half-lives or additional methods to quantify mRNA synthesis (rather than mRNA *levels*), it is not possible to determine whether early transcripts are detected during late infection due to their high stability or are transcribed throughout infection. The combined use of chemical inhibitors (e.g. cytosine arabinoside, hydroxy urea, and cycloheximide), and sampling from further time points, combined with deep sequencing would shed further light on these questions. To date no spike-in controls [70] have been included in RNA sequencing-based techniques, which would allow calculation of absolute rather than relative mRNA abundance during ASFV infection.

## ASFV promoter mapping based on mRNA 5′ termini

Beyond its use to quantify mRNA expression levels, CAGE-seq is an established technique used to map the 5′ ends of capped transcripts, thus locating transcription start sites (TSS) genome-wide [61]. Our analyses mapped TSSs for 151 of 153 ASFV-BA71V genes, as well as seven newly discovered genes [58]. Mapping of TSSs in turn allows for the identification of putative promoter elements upstream, acting as recognition motifs for transcription initiation factors. Ideally, analysis should yield distinct ASFV promoter motifs that direct differential transcription of early and late genes. However, this method is sensitive to challenges in classification of early and late genes as discussed above.

In addition to promoter mapping, TSS analyses revealed that ASFV has the potential to increase protein diversity and repertoire by using alternative (intragenic) TSSs, which result in synthesis of 5′ shortened mRNAs that encode N-terminally truncated proteins. In some cases, alternative TSS utilisation differed between early and late stages of infection. In theory, the resulting protein variants could have distinct functional properties, such as I243L, the ASFV homologue of transcription elongation factor TFIIS [47,58]. During late infection a gene internal TSS takes preference over the canonical TSS, which leads to the synthesis of a truncation variant that lacks the 52 N-terminal amino acid residues corresponding to a discrete and independently well-folded zinc-ribbon domain [71]. However, whether the truncated ASFV TFIIS variant is stable, and whether it modulates ASFV transcription in a different way than the full-length protein remains to be investigated.

## ASFV early and late promoter motifs

**Figure 3.**
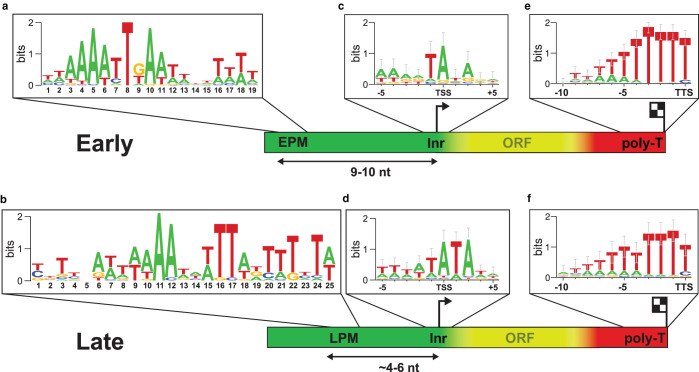
DNA consensus motifs for ASFV promoters, initiators and terminators. The sequence features of early and late gene promoter regions, including the EPM (**a**), LPM (**b**) and Inr elements (**c** and **d**). Note that spacing between the promoter motif and Inr varies in early and late gene promoters. Transcription termination is associated with a polyT signature (**e** and **f**), though a significant number of ASFV genes do not contain this termination motif hinting at an alternative, possibly factor-dependent, mode of termination.

Multiple sequence alignments of the sequences upstream of ASFV TSSs reveal two regions of conservation (Figure 3a,b). The Initiator (Inr) element is overlapping the TSS and is characterised by a very short motif, TA* and TA*TA, for early and late promoters, respectively, where the A* (asterisk) denotes the TSS (Figure 3c,d). This Inr sequence bias likely reflects the direct interactions of the single-stranded template DNA strand that is loaded into the RNAP active site. Further upstream of the TSS we identified a conserved region corresponding to the early- and late promoter motifs, EPM and LPM, respectively (Figure 3a,b). Overall, the ASFV EPM was showed a higher degree of conservation compared with the LPM, perhaps also due to a small degree of ‘contamination’ of late with early gene promoter sequences, or even a mix of intermediate and late gene promoters [58]. The EPM showed a striking similarity to the Upstream Control Element (UCE) of early VACV genes. The Vaccinia UCE binds a virus-specific heterodimeric transcription initiation factor composed of D6 and A7 [13,32,72,73], both of which are conserved in ASFV [21,74].

The questions is, which transcription initiation factors interact with the ASFV LPM? Possible candidates for post-replicative transcription initiation factors are ASFV homologues of TBP and TFIIB. In the Pol II system and archaea, these factors bind to the TATA-box and B-recognition elements, respectively, and both elements located upstream of the TSS [75–77]. This ‘ternary’ complex recruits RNAP to form the preinitiation complex (PIC). Since ASFV encodes TBP and TFIIB homologs, it stands to reason that ASFV-RNAP forms a similar PIC. However, apart from its T/A-rich nature and location upstream of the TSS, the LPM did not show significant similarity to the TATA-box consensus [53,58].

It appears likely the ASFV-D6/A7 factor binds the EPM, directing transcription from early promoters, while the virus-encoded TBP/TFIIB homologues expressed earlier during infection may be recruited to the LPM and facilitate late or post-replicative transcription. ASFV also encodes further VACV-like transcription factors which might also play a role [12]. In summary, ASFV follows at least in part the cascade-like VACV transcription paradigm [13].

## Similarity to killer plasmid promoter elements

Certain strains of yeast species, including *Klyveromyces lactis*, harbour so-called ‘Killer plasmids’ that encode a secreted toxin, providing the cell with a selective advantage in the fight for resources in their natural environment [78]. The plasmid-borne genes are transcribed by a plasmid-encoded ultra-minimal NCLDV-related RNAP system [79]. Curiously, these plasmid promoter motifs show high similarity to the VACV UCE and ASFV EPM [58,80], and the UCE-binding D6/A7 early transcription factor is not only highly conserved among NCLDVs [26], but also partially present in Killer plasmids. The D6/A7 factor has ATPase activity, and likely plays a key role in transcription of viral promoters during early infection [72,81] and, together with RNAP, contributes to the transcriptional independence from the host cell machinery. Importantly, this suggests a shared evolutionary past between viruses belonging to the NCLDV family and Killer plasmids.

## The end of transcription — ASFV termination site mapping

To gain insights into the sequence context of ASFV transcription termination, a third NGS method has been applied [58]. For ‘RNA 3′-seq’, total RNA is reverse transcribed prior to sequencing using a polyT primer that anneals to the mRNA polyA tail. The mapped reads correspond to the sequence immediately upstream of the polyA tail and uncover the native transcription termination site at nucleotide resolution [62]. This method identified transcription termination sites for more than two-thirds of ASFV genes and highlighted the presence of a ∼6–7 residue polyT (i.e. polyU in the mRNA) 3′ signature in both early and late genes (Figure 3e,f) [58]. This motif is akin to ‘intrinsic’ termination signatures in archaea [82,83] and eukaryotic Pol III [84], but differ from bacterial intrinsic terminators where the polyU stretch is preceded by RNA secondary structure elements. Approximately one third of ASFV genes did not show clear termination motifs using this method. This hints at an alternative, and perhaps factor-dependent mechanism of termination [58] that is supported by the presence of evolutionarily conserved, VACV-like, RNA helicases that could facilitate mRNA release and transcription termination [12,85].

## Untranslated regions and RNA-5′ tailing

The mapping of transcription units (i.e. regions spanning from TSS to TTS) in conjunction with well annotated ORFs (i.e. start and stop codons for protein encoding genes) enables the precise characterisation of 5′- and 3′- UTRs. The late ASFV genes had shorter 5′-UTRs with a relatively higher AT-content compared with early genes, but the functional implications of this observation are not yet clear [58]. Many ASFV mRNAs carried 5′ leaders, i.e. they included extensions at the RNA-5′ end comprised of 1–2 copies of an ‘AU’ dinucleotide. The AU and AUAU-leaders seem to be template-encoded since all mRNAs with this feature were transcribed from genes starting with the sequence A*TA corresponding to the Inr promoter motif. These extensions are thus likely generated by transcript slippage of the promoter-associated ASFV-RNAP [58]. In eukaryotes and VACV, 5′-RNA poly(A) leaders are associated with an improved translation efficiency, by circumventing the need for translation initiation factors [30,86,87]. However, whether the AUAU-leaders in ASFV have a similar function or are simply a by-product of ASFV-RNAP initiation mechanics remains to be shown.

## Host response to ASFV infection

This review has focussed on properties of the ASFV transcriptome. To elucidate the virus-host relationship, it is also important to characterise how the host transcriptome responds to ASFV infection. Only a few such studies have been published, focusing on the ASFV-GRG strain, including a total RNA-seq analysis of blood extracted from infected pigs [64], and microarray analysis of infected swine macrophage cells [88]. Both studies have provided useful insight into the host transcriptomes in response to ASFV infection, *in vivo* and *in vitro*, respectively. The RNA-seq results demonstrate that host genes involved in the immune response are up-regulated, including those associated with infection (Rtf1), monocyte macrophages (CCL5), as well as natural killer (NK) and T cells (Granzyme A). Rtf1 is linked to host transcription during adenovirus infection [89], Granzyme A is a proapoptotic protease [90], and CCL5 expression increases during early ASFV infection [91,92]. This trend was also reflected in KEGG pathway analysis of DEGs, which found up-regulation of cytokine, NK cell and RIG-I-like receptor signalling pathways. Similarly, immune response-related pathways were up-regulated in a microarray analyses, including DEGs in the RIG-I-like receptor pathway, NK cell mediated cytotoxicity, and multiple pathways associated with T cells or type I interferon (IFN) signalling [93]. IFN signalling is an essential aspect of host innate and adaptive immune responses to viral infection [94], making it understandable components of IFN signalling pathways were found up-regulated in both gene expression studies of ASFV-infected hosts. ASFV does, however, respond to the host immune system with its own arsenal of countermeasures. These include proteins inhibiting apoptosis and IFN, also preventing self-inhibition of host protein synthesis. However, many of these ASFV genes can be deleted without hindering viral replication, suggesting ASFV uses a range of complementing countermeasures that are still being uncovered [95].

## Discussion

Outbreaks of ASF among domestic and wild pigs are spreading rapidly in Africa, Europe and Asia, with devastating economic impact in affected countries. This is the most significant animal disease in recent history: it has a mortality rate of up to 100% and many alternative routes for viral spread, including direct contact, contaminated feed or equipment, and transmission via soft tick vectors. Currently, there is no implemented or approved vaccine for ASF, and the fight against it is limited to physical sanitation and control measures, as well as culling of infected animals (World Organisation for Animal Health, www.oie.int) [2–6].

Apart from the socioeconomic importance of ASFV, its viral transcription system is of interest among the transcription community, especially for those working on the structure, function and evolution of multisubunit RNA polymerases, as ASFV-RNAP represents a streamlined version of Pol II (Figure 1) [12]. Transcriptomics approaches are invaluable for analysing viral transcription, as well as the host response to infection — and the interplay between virus and host gene expression (Figure 2) [58,64,93]. Lastly, understanding molecular mechanisms of ASFV transcription, including regulatory DNA sequences (Figure 3), factors involved, and temporal gene expression patterns is essential to devise an efficient response to the virus, with the aim of developing antiviral drugs and efficient vaccines.

Going forward the important task at hand is to study and characterise the molecular mechanisms underlying ASFV transcription. This includes (i) carrying out further transcriptome analyses using combinations of chemical inhibitors in order to unequivocally determine all ASFV gene expression stages. Thus, extending our understanding of how viral transcription initiation factors and cognate promoters co-operate to enable gene expression during early and late infection, as well as investigate whether host factors participate in ASFV transcription. Likewise (ii), an essential step for understanding these mechanisms requires determining the whole-genome distribution of ASFV-RNAP and initiation factors, to study transcription complexes throughout infection. Lastly (iii), it is additionally important to produce recombinant RNAPs and transcription factors for structural characterisation, and *in vitro* transcription assays, to biochemically characterise ASFV transcription. Allowing also, for testing of chemical compound libraries for ASFV-RNAP inhibitory activity, and therefore antiviral drugs.

## Perspectives

- Importance for the field: Outbreaks of African Swine Fever are spreading rapidly throughout Eastern Europe, Africa and Asia, making the threat of ASFV of critical socioeconomic importance for agriculture and pig farming industries worldwide. With disease control measures (such as culling) being the only available option, since there is no approved vaccine for this disease with a mortality rate up to 100% (World Organisation for Animal Health, www.oie.int).
- Current thinking: Transcriptomics have provided essential insight into understanding mechanisms underlying ASFV gene expression and its interplay with that of the host. Specialised next generation sequencing techniques have also been used to map the 5′ and 3′ ends of ASFV transcripts genome-wide, which has enabled identification of promoter and transcription termination motifs, respectively, expanding our understanding of how ASFV modulates its gene expression.
- Future directions: Moving forward, work should focus on further transcriptomics to discern other viral gene expression stages and their respective promoters, complemented by whole-genome distribution studies of ASFV-RNAP and transcription factors. Additionally, production of these proteins recombinantly, will allow for their structural characterisation and functional assessment with biochemical assays, thus enabling screening for compounds showing viral transcription inhibition and therefore could act as anti-viral drugs.

## Open Access

Open access for this article was enabled by the participation of University College London in an all-inclusive *Read & Publish* pilot with Portland Press and the Biochemical Society under a transformative agreement with JISC.

## Abbreviations

ASF: African Swine Fever
ASFV: African swine fever virus
CAGE-seq: Cap Analysis Gene Expression sequencing
DEGs: differentially expressed genes
IFN: type I interferon
NCLDVs: Nucleocytoplasmic large DNA viruses
NK: natural killer
PIC: preinitiation complex
TBP: TATA-binding protein
TSS: transcription start sites
UCE: upstream control element
UTR: untranslated region
VACV: Vaccinia virus
